# Microleakage Comparison of Four Dental Materials as Intra-Orifice Barriers in Endodontically Treated Teeth

**Published:** 2012-03-01

**Authors:** Hamid Reza Yavari, Mohammad Samiei, Shahriar Shahi, Mohammad Aghazadeh, Farnaz Jafari, Majid Abdolrahimi, Saeed Asgary

**Affiliations:** 1. Department of Endodontics, School of Dentistry, Tabriz University of Medical Sciences, Tabriz, Iran; 2. Department of Microbiology, School of Medicine, Tabriz University of Medical Sciences, Tabriz, Iran; 3. Private Practice, Tabriz, Iran; 4. Iranian Center for Endodontic Research, Dental Research Center, Shahid Beheshti University of Medical Sciences, Tehran, Iran

**Keywords:** Amalgam, Calcium Enriched Mixture, Dental Leakage, Endodontics, MTA, Saliva

## Abstract

**Introduction:**

The aim of this in vitro study was to compare polymicrobial microleakage of calcium enriched mixture (CEM) cement, mineral trioxide aggregate (MTA), amalgam, and composite resin as intra-orifice sealing materials.

**Materials and Methods:**

Seventy single-rooted mandibular premolars were instrumented and obturated by cold lateral compaction technique. The teeth were randomly divided into four experimental groups according to used material: CEM, MTA, amalgam and composite resin (n=15) and two control groups (n=5). In experimental groups, 2 mm of coronal gutta-percha was removed and replaced with the study material. All the teeth were mounted in a two-chamber apparatus and the coronal portion was exposed to human saliva. The day the turbidity occurred was recorded for each sample. Data were analyzed using one-way ANOVA.

**Results:**

The negative control group showed no leakage while the average microleakage time in the positive control group was 3.5 days. The average bacterial leakage times for amalgam, composite resin, MTA, and CEM groups were 27.42±3.6, 29.35±3.15, 52.57±2.87, and 50.42±2.73 days, respectively. There was no significant difference between CEM and MTA groups (P=0.27) and also between amalgam and composite resin groups (P=0.36). However, in term of average leakage time, MTA and CEM groups exhibited significant differences with amalgam and composite resin groups (P<0.001).

**Conclusion:**

According to the results of the present in vitro study, in terms of coronal sealing in endodontically treated teeth, CEM and MTA are more effective than amalgam and composite resin.

## Introduction

Secondary microleakage due to compromised coronal seal is one of the most important factors associated with endodontic treatment failures. According to Tselnik et al. insufficient coronal seal may occur in different clinical situations, like fracture of tooth structure, missing of temporary filling materials, marginal leakage of the final restoration and recurrent caries. All these conditions expose the root canal system to the oral environment with subsequent coronal microleakage [1].

Intra-orifice barrier is an efficient alternative method to decrease coronal leakage in endodontically treated teeth. This procedure includes placing additional material into the canal orifices immediately after removal of the coronal portion of gutta-percha and sealer [2]. Several materials have been used in an attempt to provide an intra-coronal seal to prevent microleakage, such as Cavit, amalgam, intermediate restorative material (IRM), Super-EBA, composite resin, glass-ionomer cement and mineral trioxide aggregate (MTA) [3][4]. Based on the results of a study by Roghanizad and Jones, the sealing ability of amalgam as an intra-orifice barrier is significantly better than those of Cavit and TERM [2][5]. Ferk et al. showed that poly-microbial microleakage of MTA is less than that of amalgam in a simulated coronal leakage model [5].

MTA is a biomaterial introduced for endodontic applications during the early 1990s. MTA is derived from Type I Portland cement and is composed of dicalcium silicate, tricalcium silicate, tricalcium aluminate, tetracalcium aluminoferrite, and bismuth oxide [6]. It has numerous clinical applications such as pulp capping, pulpotomy, treatment of internal root resorption, undeveloped apices (apexogenesis and apexification), root-end filling, repair of root and furcation perforations [7][8], and also as a coronal barrier [1]. In the majority of studies, MTA has exhibited better microleakage protection than conventional endodontic materials using various methods [9]. On the other hand, long setting time [6], poor handling [10], and relatively high price are some of its disadvantages.

Recently, calcium enriched mixture (CEM) cement has been introduced to endodontics. It consists of different calcium compounds which provide a bioactive calcium- and phosphate-enriched material when being mixed with a water-based solution; CEM biomaterial can set and be used in an aqueous environment, with having good handling properties and reasonable price [11][12][13]. In microleakage studies it has been shown that sealing properties of CEM cement are comparable to those of MTA when being used as a root-end filling material [14][15].

According to the results of various studies, the use of polymicrobial analysis of micro-leakage to evaluate leakage is of higher biological and clinical relevance than other assessment methods, such as dye leakage, fluid filtration and glucose leakage model [1][3][5][16][17][18][19][20]. Different of studies have been carried out on coronal sealing ability of MTA and other restorative materials; however, there is no such research on CEM cement. Therefore, the purpose of this in vitro study was to compare the coronal sealing properties of CEM cement, MTA, amalgam and composite resin by human saliva microleakage model in endodontically treated teeth.

## Materials and Methods

In this experimental study, 70 freshly extracted caries-free single-rooted human mandibular premolars were used. The teeth were examined under a light stereomicroscope to make sure they did not have any cracks. All teeth were decoronated with a high-speed handpiece under copious water cooling to provide identical 11±0.5-mm roots. The root canals were prepared with K-files #15, #20, and #25 (Dentsply, Maillefer, Ballaigues, Switzerland) 1 mm short of the apex, followed by RaCe rotary files (FKG, La-Chaux De Fonds, Switzerland) #0.10/40, #0.08/35 and #0.06/30, using crown-down technique.

Irrigation was carried out with 1% sodium hypochlorite (NaOCl) during preparation. Finally, the canals were irrigated with 17% EDTA (Diadent Inc, Chongchong Buk Do, Korea) to remove the smear layer, followed by irrigation with 5 mL of normal saline. All the specimens were checked again for cracks under a light microscope. Roots with cracks were discarded and replaced.

After drying with sterile paper points (Ariadent, Tehran, Iran), the root canals were obturated with gutta-percha (Ariadent, Tehran, Iran) and AH26 sealer (DeTrey, Dentsply, Konstanz, Germany) using lateral compaction technique. The coronal 2-mm of all the canals was emptied with a heat carrier and gutta-percha was vertically condensed by a plugger. A probe was used to control the depth of the intra-orifice cavity. Excess sealer of the dentinal walls was removed with alcohol-soaked cotton pellets.

The teeth were randomly divided into four experimental groups (n=15): ProRoot MTA (Tooth-colored Formula, Dentsply, Tulsa Dental, Tulsa, OK), amalgam (Non-gamma-2 Admix Amalgam, SDI Limited, Australia), flowable composite resin (Filtek Flow, 3M ESPE, St. Paul, MN, USA) with Single Bond (Single Bond, 3M ESPE, St. Paul, MN, USA),and CEM cement (BioniqueDent, Tehran, Iran) and also 2 positive and negative (without a coronal barrier material; n=5) control groups.

The experimental (bio)materials were used according to manufacturer’s instructions. Then the specimens were radiographically examined for the length and density of the sealing material. In the positive control group no sealing material was used. The teeth were kept at 37°C and 95% relative humidity for 7 days. In the next step, two layers of nail varnish were placed on all the root surfaces except for the apical 2 mm and the coronal plane. In the negative control group all the root surfaces were covered.

All the specimens were mounted in a saliva microleakage assessment apparatus. First, the roots were placed in 1.5 mL plastic Eppendrof (Elkay, Shrewbury, MA, USA) tubes. Connection areas were sealed with two layers of cyanoacrylate adhesive. The whole system was sterilized with ethylene oxide gas for 12 hours and then placed in sterile glass flasks containing 6 mL of sterile BHI (BHI-Oxide LTD, Hanks, USA) while the apical 2 mm of the root apices were immersed in the broth.

The samples were incubated for 7 days at 37°C to make sure of the sterilization process; lack of turbidity ensured sterility of the setups. Then fresh saliva was added into the upper parts of the tubes, which were refilled by the same person’s saliva every day. All the specimens were kept at 37°C and lower parts of the tubes were checked on a daily basis for color changes and turbidity, which would indicate bacterial growth.

When a BHI showed color changes, evaluation of that sample was terminated and the turbid solutions were labeled “microleakage positive”. The day the turbidity occurred was recorded for each sample. The whole system was incubated for 90 days.

In order to evaluate the validity of bacterial leakage, saliva and turbid BHI solutions were incubated in blood agar plates for 18 to 25 hours and morphological characteristics and hemolysis behavior of the colonies were studied. All the statistical evaluations were carried out using one-way ANOVA and a post hoc Tukey test. Statistical significance was set at P<0.05.

## Results

### Saliva leakage time

The average bacterial leakage time for amalgam, composite resin, MTA and CEM were 27.42±3.6, 29.35±3.15, 52.57±2.87, and 50.42±2.73 days, respectively. The negative control group showed no leakage until the end of the experimental period, while the average leakage time in the positive control group was 3.5 days.

**Figure 1:**
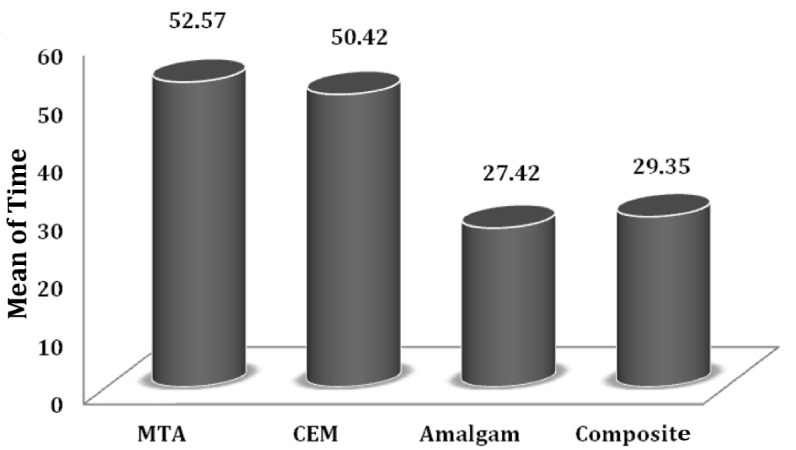
Mean time (Days) for microleakage to occur in the four experimental groups

One-way ANOVA showed statistically significant differences in average leakage time of the four experimental groups. Post hoc Tukey test results revealed that microleakage of MTA was significantly different in comparison to amalgam and also composite resin groups (P<0.001). On the other hand, the average microleakage of CEM cement had significant differences with amalgam and composite resin groups (P<0.001), while there was no significant differences between MTA and CEM cement groups (P=0.27) and also amalgam and composite resin groups (P=0.36) in mean leakage times (Figure 1).

### Results of microbial culture

Microbial analysis of cultured saliva in blood agar plate was Staphylococcus, Streptococcus, Dyphtheroids and Niesseria sica. Microbial analysis of turbid BHI solution in blood agar plate was Staphylococcus, Streptococcus and Dyphtheroids.

The samples from the lower part of the apparatus, in which no turbidity appeared, such as negative control group specimens, did not show any bacterial growth.

## Discussion

Coronal microleakage is an important factor in endodontic failure [21]. Conventional root filling materials such as gutta-percha and sealer provide minimal resistance to bacterial micro-leakage [22][23]. Numerous materials have been investigated as coronal sealants; however, they have shown various degrees of leakage [4][24][25]. Therefore, attempts are underway to introduce more qualified materials with the potential to provide a long-term seal.

The use of bacteria to evaluate apical, and mainly coronal leakage, is considered to be of greater clinical and biological relevance than other leakage assessment methods [26]. Usually special species or a limited number of bacteria are used for this method; hence, bacterial synergistic effect, influence of environment thermal changes, salivary enzymes, buffering materials and antibodies are neglected [20][27][28][29]. Polymicrobial analysis of microleakage is considered the best method because it simulates clinical situations of the oral environment [5][16][17][22]. Therefore, some researchers have used multi-species method with artificial saliva [5][17][22] and some have used whole human saliva [18]. Because of the close relationship with natural oral conditions, the model used in this study was polymicrobial comparison of coronal microleakage with fresh human saliva.

The results of a study carried out by Roghanizad and Jones revealed that amalgam, as an orifice plug, is more efficacious than Cavit in preventing coronal microleakage [2]. Tselnik et al. reported no differences in bacterial penetration with human saliva between gray MTA, white MTA, or a resin-modified glass-ionomer restorative material [1]. According to the results of a study carried out by Feric Luketic et al. MTA is considerably better than amalgam as an intra-orifice barrier [5], which is consistent with the results of the present study. Based on the findings of the recent study, using the glucose penetration model, Sanchez et al. reported that CavitTM G, Tetric EvoFlowor and ProRoot MTA in the testing period had similar leakage resistance abilities when used as intra-orifice barriers [29].

Barrieshi-Nusair and Hammod compared glass-ionomer and MTA as orifice plugs and reported that glass-ionomer has more micro-leakage [30] but to date CEM cement has not been studied as an intra-orifice plug while it has been used for treatment of furcal perforations, vital pulp therapies in permanent and primary teeth, root-end filling, management of root resorption, and revascularization for necrotic immature permanent molars [12][13][31][32][33][34][35][36][37][38][39][40]. Therefore, we decided to compare the coronal microleakage of CEM cement with that of amalgam, composite resin and MTA by a relatively valuable microleakage comparison model.

In the present study the highest turbidity average time was observed in the MTA and CEM cement groups and the lowest was noted in the positive control group with an average of 3.5 days, demonstrating significant differences. The important point is that the teeth with CEM cement or MTA coronal seal have better protection against microbial leakage in comparison with the teeth without coronal seal during the test period.

Several studies compared the sealing properties of CEM cement with MTA as root-end filling materials using dye/bacterial penetration methods; the results showed that MTA and CEM cement groups created favorable apical/coronal seal [15][41][42][43][44]. These results are concurring with the present finding which assessed the sealing potential of these biomaterials as intra-orifice plugs.

The results of the present study revealed that CEM cement’s potential as an intra-orifice barrier against bacterial penetration is comparable with that of MTA and higher than that of amalgam and composite resin. The potential of these two biomaterials in preventing bacterial leakage as canal orifice barriers is comparable. These favorable sealing properties, in most part, are related to hydrophilic nature, good anti-bacterial/fungal potential, high pH and formation of hydroxyapatite crystals in MTA and CEM cement materials [11][45][46][47][48].

## Conclusion

According to this in vitro study, we can conclude that CEM cement and MTA, as intraorifice sealing bio-materials, are more effective than amalgam and composite resin in preventing saliva leakage in endodontically treated teeth.
